# A second generation cervico-vaginal lavage device shows similar performance as its preceding version with respect to DNA yield and HPV DNA results

**DOI:** 10.1186/1472-6874-13-21

**Published:** 2013-05-02

**Authors:** Viola MJ Verhoef, Maaike G Dijkstra, Remko P Bosgraaf, Albertus T Hesselink, Willem JG Melchers, Ruud LM Bekkers, Johannes Berkhof, Folkert J van Kemenade

**Affiliations:** 1 Department of Pathology, VU University Medical Center, (VUmc) De Boelelaan 1117, Amsterdam 1007 MB, The Netherlands; 2 Department of Obstetrics and Gynaecology, Radboud University Nijmegen Medical Centre, Nijmegen, The Netherlands; 3 Department of Medical Microbiology, Radboud University Nijmegen Medical Centre, Nijmegen, The Netherlands; 4 Department of Epidemiology and Biostatistics, VU University Medical Center, Amsterdam, The Netherlands

**Keywords:** Human papillomavirus, Self-sampling, HPV DNA testing, Cervical screening, Self-sampling device

## Abstract

**Background:**

Attendance rates of cervical screening programs can be increased by offering HPV self-sampling to non-attendees. Acceptability, DNA yield, lavage volumes and choice of hrHPV test can influence effectiveness of the self-sampling procedures and could therefore play a role in recruiting non-attendees. To increase user-friendliness, a frequently used lavage sampler was modified. In this study, we compared this second generation lavage device with the first generation device within similar birth cohorts.

**Methods:**

Within a large self-sampling cohort-study among non-responders of the Dutch cervical screening program, a subset of 2,644 women received a second generation self-sampling lavage device, while 11,977 women, matched for age and ZIP-code, received the first generation model. The second generation device was different in shape, color, lavage volume, and packaging, in comparison to its first generation model. The Cochran’s test was used to compare both devices for hrHPV positivity rate and response rate. To correct for possible heterogeneity between age and ZIP codes in both groups the Breslow-Day test of homogeneity was used. A T-test was utilized to compare DNA yields of the obtained material in both groups.

**Results:**

Median DNA yields were 90.4 μg/ml (95% CI 83.2-97.5) and 91.1 μg/ml (95% CI 77.8-104.4, p= 0.726) and hrHPV positivity rates were 8.2% and 6.9% (p= 0.419) per sample self-collected by the second - and the first generation of the device (p= 0.726), respectively. In addition, response rates were comparable for the two models (35.4% versus 34.4%, p= 0.654).

**Conclusions:**

Replacing the first generation self-sampling device by an ergonomically improved, second generation device resulted in equal DNA yields, comparable hrHPV positivity rates and similar response rates. Therefore, it can be concluded that the clinical performance of the first and second generation models are similar. Moreover, participation of non-attendees in cervical cancer screening is probably not predominantly determined by the type of self-collection device.

## Background

In developed countries, incidence rate of and mortality from cervical cancer have decreased since the introduction of cytology based cervical screening [1-4]. However, randomized controlled trials have shown that high-risk human papillomavirus (hrHPV) testing provides a superior protection against high-grade cervical intraepithelial neoplasia than cytology [5-7]. Therefore, population based screening could be improved by the introduction of primary hrHPV testing. However, this will not affect the participation rate of programmed cervical screening, which often is suboptimal. Since non-attendance is associated with an increased risk of developing cervical cancer, it is especially important to reach these non-attending women [1,8,9]. Recent studies have shown that offering self-collection devices for hrHPV testing on cervico-vaginal specimens to non-attendees may improve compliance to screening [10-17]. Additionally, self-sampling has facilitated access to cervical screening for women in developing countries [18-20].

Meta-analyses and systematic reviews have shown a high level of concordance in HPV detection rates between self-sampled specimens and clinician-collected samples [21-24]. Moreover, some studies have reported a similar sensitivity between the two sampling methods with respect to detection of high-grade cervical intraepithelial neoplasia or worse (CIN2+), but data are inconsistent [21-23,25-27]. The variations in clinical performance might be explained by the use of different HPV assays in combination with various self-sampling devices (e.g., brushes, swabs, tampons and lavage devices) [27]. Nevertheless, offering a self-sampling device seems a good method to increase cervical screening coverage [11].

An example of a frequently used self-collection method which can rinse the upper vagina and cervix to obtain cervico-vaginal material is the Delphi lavage device® (Delphi Bioscience BV, Scherpenzeel, The Netherlands). Brink et al. [25] validated the first generation of this device by showing that its use, in combination with hrHPV testing by GP5+/6+ PCR, resulted in a similar detection rate of CIN2+ lesions compared to physician sampling of cervical material. This device has successfully been used in studies with women who did not respond to an invitation of the regular cervical screening program [11,13,14].

Yet, based on spontaneous and actively requested feedback from more than 10,000 study-participants as well as market research, a modification of this lavage sampler (‘second generation’) was introduced in order to increase user-friendliness (see Methods section for the alterations). A questionnaire-based Dutch study scoring for first impression, showed higher scores for the second generation device (n=155) when compared to scores of women in a separate study using the first generation device (unpublished data). A user questionnaire study on the use of the second generation device (n=50) showed a high acceptability and user-friendliness (measured for ease of its use, ease to follow instructions, comfort level, recommendability and preferability of device for the next screening) comparable to the New York study among 197 women [28,29]. So the second generation device showed an improved first impression compared to the first generation and a negligible effect on user acceptability which was already high.

**Figure 1:**
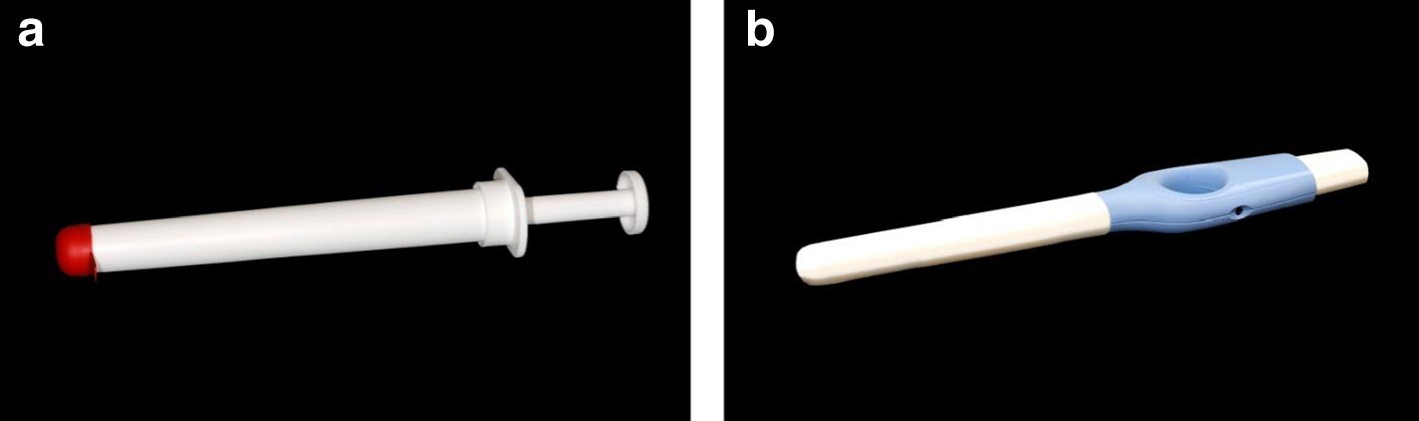
**Design of the Delphi Screener. a**: First generation of the device **b**: Second generation of the device.

This study compares clinical performance of the second generation model of a self-sampling device (Figure 1b) to the first generation model (Figure 1a) by comparing the hrHPV positivity rate, acquired DNA yields and participation rate.

## Methods

### Study population

Our validation study was conducted within a large study among non-attendees of the screening program in the year 2007, which were offered self-sampling between October 2010 and June 2011. This trial was approved by the national ethics committee (Ministry of Public Health No 2010/04WBO) and informed consent was given by all participating women. From this large study we excluded women whose age and ZIP-code combination was not observed in both subgroups (of women who received the second generation sampler). In total, we retained 2,644 women who had received the second generation sampler and 11,977 women who had received the first generation of the device. All participants received a self-sampling kit at their home address with a collection device, a collection tube, an explanatory letter, an informed consent form, user instructions and a return envelope. Women were asked to return the collection tube containing the cervico-vaginal lavage specimen and the signed informed consent form in a return-envelope to the laboratory of the VU University Medical Center for hrHPV testing. In both groups, self-sampling kits contained similar content except for the version of the lavage device and the accompanying user instructions.

### Sampling

The first generation sampler is a plastic, syringe-like device and is provided in a blister. The sampler is pre-filled with 5 ml sterile saline. The handgrip, as well as the insertion part are white colored and the diameter of the insertion part is 1.8 cm. The top of the insertion part is covered with a red silicone cap that prevents the fluid from leaking and prevents accidental use of the plunger. Once the cap is removed, the plunger can be pushed (See Additional file 1 for user instructions of the first generation device).

The second generation sampler is a plastic device provided in a pouch. The sampler is pre-filled with 3 ml sterile saline, which reduces the risk to leak lavage fluid during sample collection compared to the first generation. The insertion part itself did not change in length, as 12 cm is necessary to reach the cervix in the majority of women. However, the insertion part of the second generation device is distinguished from the handgrip by color (white and blue respectively). The diameter of the insertion part is 1.6 cm; therefore, this new model is leaner than the first generation. The top of the insertion part is covered with a seal to prevent leakage and accidental use and is easier to remove than the cap of the first generation device. Instead of a syringe-like mechanism for which the thumb is needed to push the plunger, the second generation is designed to improve both the grip and strength to push the plunger. Moreover, the new design can be used in laying down as well as in sitting position. See Additional file 2 for user instructions of the second generation device.

### HPV testing

The self-sampled material was tested for hrHPV in two different laboratories, respectively the department of Pathology, VU University Medical Center (VUmc), Amsterdam, the Netherlands and the department of Medical Microbiology, Radboud University Nijmegen Medical Centre (RUNMC), Nijmegen, the Netherlands. Upon arrival of the self-sampled material in the laboratory, tubes were centrifuged to concentrate the cell material from the lavage specimens. Subsequently, the supernatant was removed and the pellet was resuspended in 1.5 ml ThinPrep preservation medium (Hologic). The Hamilton MICROLAB STARlet robot (VUmc) and the Roche MagNA Pure LC Isolation station (Roche Diagnostics) (RUNMC) were used to isolate DNA from 1/10^th^ of this material. The hrHPV test was performed on the isolated material by the GP 5+/6+ PCR-EIA, as described previously [30]. Furthermore, for quality control for the presence of DNA and the absence of PCR inhibitors in the isolated material, a PCR for the B-globin gene was performed on the isolated material of those samples that had a visually small pellet at arrival in the lab, and on another random 11% of the samples. In case of hrHPV positive material, DNA yield (which is a proxy for the cell yield) was estimated by measuring DNA concentrations (ng/μl) in 1 μl DNA extract using the NanoDrop (Thermo Scientific) according to recommendations of the manufacturer. This value was subsequently used to calculate the DNA yield (μg/mL) in self-sampled material.

### Statistical analysis

To test whether the hrHPV positivity and participation rate differed between a first and second generation device, women were stratified according to ZIP code and age (5 year cohorts). Strata without data for both the first and second generation device were discarded. Note that the hrHPV positivity and participation rate have different denominators and hence the number of strata may be different for the hrHPV positivity and participation rate analyses. To check for possible heterogeneity between age and ZIP codes, the Breslow-Day test of homogeneity was used. Within strata, differences between the first and second generation devices were tested by Mantel Haenszel Cochran’s test. A T-test was utilized to compare DNA yields of the obtained material in both groups. A logistic regression was performed to estimate the effects of age on response rate, separately for the first and second generation of lavage device.

## Results

### Characteristics of trial cohort

**Figure 2:**
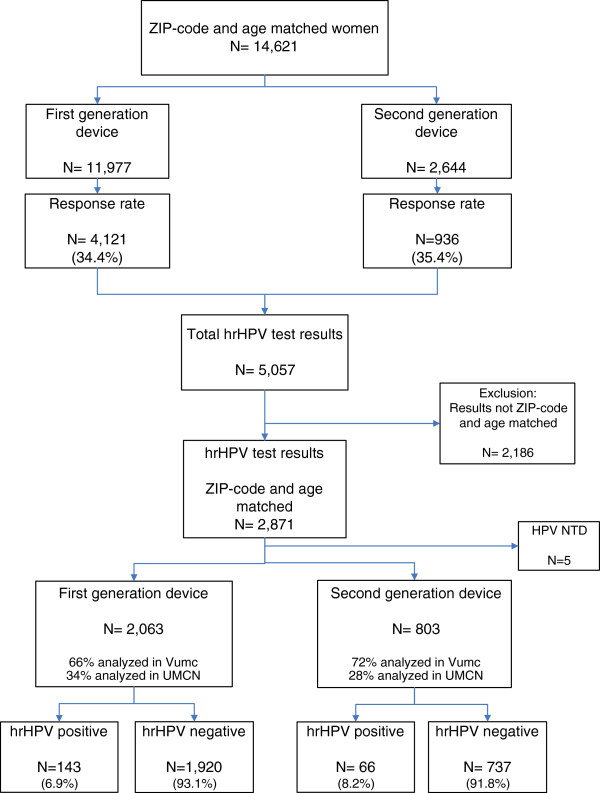
Study design.

The flowchart of the study is shown in Figure 2. The total study group involved 14,621 women between 33 and 63 years old (median age of 43.4 years) of whom 2,644 women (18.1%) received the second generation of the device and 11,977 (81.9%) ZIP code and age-matched women received the first generation.

### HrHPV detection rate

Of all 14,621 invited women, 5,057 participated by returning their material to our laboratory for hrHPV testing. For our comparison of the hrHPV detection rate, 2,871 women were taken into account of whom the age and ZIP-codes matched between both subgroups. Five women were excluded because of an invalid hrHPV test due to a negative result of the quality control; the results of this quality control are described in the next paragraph. Thus, in total, the self-samples of 2,866 women were taken into account to calculate and compare the hrHPV positivity rates. Of the 803 participants in the second generation group who submitted a self-sampled specimen, 66 women (8.2%) were tested hrHPV positive (Table 1), while the first generation group included 143 (6.9%) hrHPV positive women. The hrHPV positivity rate was not significantly different between the two generations (OR 1.123, 95% CI 0.813-1.552, p= 0.419).

**Table 1:** hrHPV positivity rate of the second generation of the device versus the first generation

		**hrHPV test result**	
		**hrHPV negative**	**hrHPV positive**^**a**^
**Type of Screener**	**First generation**	1,920 (93.1%)	143 (6.9%)
	**(control)**		
	**Second generation**	737 (91.8%)	66 (8.2%)
	**(case)**		
	**Total**	**2,657 (92.7%)**	**209 (7.3%)**

^a^ p= 0.419 (Cochran’s test).

### Quality control

A random quality control, consisting of a random B-globin PCR, as performed on 315 of the 2,871 samples, has revealed that there is no difference between the assay performance on the first compared to the second generation device. Two out of 234 samples (0.9%) collected by the first generation device had a negative B-globin result. None of the 81 samples obtained with the second generation device were B-globin negative. In addition to the random quality control, we performed a quality control on all samples with a visually small pellet by arrival in our laboratory (n=56). Of the 33 samples with a visually small pellet that were collected by the first generation of the device, one sample (3.0%) had a negative B-globin test. In samples collected by the second generation device, two of the 23 checked samples (8.7%) were B-globin negative. The five samples with a negative B-globin test were scored as ‘invalid HPV test result’ because of insufficient material, while all samples with a positive B-globin test were scored as ‘sufficient material’ and were taken into account for further analysis with regard to the hrHPV detection rate as described above.

### DNA yield

DNA yields were measured in all hrHPV positive samples of the total participants’ cohort of 5,057 women (Figure 2). These samples comprised 81 samples collected with the second generation of the device and 295 with the first generation. The mean DNA yield (μg) per ml sampled material with the new version was 91.1 μg/ml (95% CI 77.8-104.4), and that of material sampled with the earlier version was 90.4 μg/ml (95% CI 83.2-97.5) per sample (Table 2). The T-test was used to compare these DNA yields. The distribution of the lnDNA concentrations was confirmed to be normal. The T-test showed that an equal DNA yield was obtained with both self-sampling devices (p= 0.726). The mean difference between the two sampling methods was 1.039 (95% CI 0.173-1.286).

**Table 2:** Comparison of DNA concentration of the second generation versus the first generation in hrHPV positive samples

**DNA yield in μg per ml self-sampled material**		
	**First generation (n=295)**	**Second generation (n=81)**
**Mean**^**a**^	90.4	91.1
	(95% CI 83.2-97.5)	(95% CI 77.8-104.4)
**Median**	79.4	79.7
**Minimum**	1.0	3.1
**Maximum**	391.2	256.5

^a^ p= 0.726 (T-test).

### Participation rate

Table 3 shows the participation rate of all women in the study. In the group of women who received the second generation, 936 of the 2,644 invited women (35.4%) participated by sending their self-sampled specimen to the laboratory. In the cohort that received the earlier version, 4,121 of the 11,977 women (34.4%) returned their self-sampled material. Taken together, a similar participation rate was observed (OR 1.020, 95% CI 0.930-1.118, p= 0.654). In Table 4, all invited women were grouped into seven age cohorts to measure response rate for each age group. In none of the age groups there was a difference in response rate in women who received the first generation device compared to those who received the second generation (i.e., all Pearson chi-square p values were >0.05). Furthermore, a logistic regression analysis was performed to measure whether the response rate was associated with age. This analysis showed no effect of age neither for the first nor for the second generation device. Odds ratios were 0.982 (95% CI 0.960-1.004, p= 0.112) and 0.992 (95% CI 0.948-1.039, p= 0.745), respectively.

**Table 3:** Participation rates in women receiving the second generation and the first generation of the device

		**Response rate**	
		**No response**	**Response**^**a**^
**Type of Screener**	**First generation**	7,856 (65.6%)	4,121 (34.4%)
	**(control)**		
	**Second generation**	1,708 (64.6%)	936 (35.4%)
	**(case)**		
	**Total**	**9,564 (65.4%)**	**5,057 (34.6%)**

^a^ p= 0.654 (Cochran’s test).

**Table 4:** Comparison of participation rates by age group in women receiving the second generation and the first generation of the device

	**Response rate**				
**Age group (in years)**	**First generation device**		**Second generation device**		
	**n**	**%**	**n**	**%**	**p-value**^**a**^
**60-64**	71	25.9%	26	33.8%	**0.173**
**55-59**	330	32.0%	85	32.4%	**0.901**
**50-54**	519	35.8%	141	38.6%	**0.323**
**45-49**	564	34.1%	122	32.0%	**0.435**
**40-44**	706	34.3%	169	37.1%	**0.250**
**35-39**	1,085	36.3%	213	36.8%	**0.836**
**30-34**	846	33.5%	180	34.3%	**0.717**
**Total**	**4,121**	**34.4%**	**936**	**35.4%**	

^a^ p values in all age groups are calculated by the Pearson Chi-Square test.

## Discussion

Our data show that both the hrHPV positivity rate and the DNA yield of the second generation of the Delphi screener were equal to those of the validated, first generation of this device. With regard to the hrHPV positivity rate, this study found comparable rates in women who received the second generation (8.2%) compared to women in an age- and ZIP code matched group who received the first generation (6.9%), while several other studies have shown different percentages; it is likely that these variations are caused by different study populations, different devices and HPV tests that were used within studies [11,14,26,27]. The hrHPV positivity rates in our study are in line with some other reported hrHPV positivity rates among non-attendees [12,16].

In addition, this study showed that the DNA concentration of hrHPV positive samples collected with the second generation was comparable to the mean concentration in samples obtained with the validated, first generation. This is interesting, because the lavage volume of the second generation of the device is smaller (3 ml vs. 5 ml in the first generation). Since a similar DNA concentration was measured in samples obtained with this second generation, these samples still seem appropriate for testing other molecular (triage) markers, such as methylation markers, on the self-sampled material, [31] (Hesselink et.al., manuscript in preparation).

Manufacturing and logistic cost saving advantages of the second generation device over the first generation are reported. First, the design of the second generation device enables high volume production with lower cost price. Second, the smaller diameter and packaging of the second generation device allows sending by mail while the first generation had to be hand-delivered by a postman at a higher rate.

The attendance rate in this study was 34.4% with the first generation and 35.4% with the second generation. A previous study, in which non-attendees received the first generation of the Delphi Screener, showed a slightly lower attendance rate of 27.5% [11]. Two other studies among non-attendees of the Dutch screening program, in which women were invited to take a brush-based vaginal self-sample, showed a response rate of 34.2% and 30.8%, respectively [10,12]. The attendance rate differs only slightly in these studies, possibly either due to the type of self-sampling device or because women had prior knowledge of self-sampling. Cohort effects, including seasonal influences, could also play a role. In this study, a possible effect of cohorts was minimized because the samples were tested within one cohort.

An important limitation of the current study is that we did not have histological follow-up of the participating women yet to compare the clinical accuracy of the different versions of the self-sampling device. However, we did find an equal hrHPV positivity rate and DNA concentration; therefore, we expect that a comparable percentage of CIN2+ lesions can be identified independent of the model that was used, and this study therefore bridges the studies with the earlier device [11,25] to future studies with the second generation device. Furthermore, inter-laboratory heterogeneity could have affected our hrHPV test results, because two different laboratories performed the HPV tests in our study. However, we have automatically corrected for this type of bias by correcting for ZIP code by using a posterior matching procedure, as samples from women within the same ZIP code were always analyzed in the same laboratory. The strengths of our study are that we collected our data in a nested case–control study within similar age cohorts, and matched for ZIP codes and age distribution within the cohorts [32].

## Conclusions

This study shows that offering a new and improved second generation of the cervico-vaginal lavage device results in a comparable hrHPV positivity rate and that samples contain the same DNA yield as compared to the first generation of this device. In addition, a comparable small number of invalid samples were observed taken by the second generation of the device compared to the first generation. Therefore, it can be assumed that this second generation device can be employed with similar reliability as the first generation device to improve attendance among non-responders in screening programs. The new design of the device offers cost saving production and logistic advantages while it seems to have similar effect on the participants’ response rate.

## Competing interests

HB has received speakers’ fee from Qiagen. None of the authors received reimbursements, fees, funding, salary, or hold any stocks or shares in an organization that may gain or lose financially from the publication of this manuscript. None of the authors hold patents or are currently applying for any patents relating to the content of the manuscript. None of the authors have any non-financial competing interests.

## Authors’ contributions

VV and FvK managed the study. VV and RBo did the acquisition of the data. VV, MD and FvK drafted the manuscript. VV and JB performed the statistical analysis. AH, RBo and WM were responsible for the HPV testing. AH analyzed the cell yield measurements. RBe liaised with the gynaecologists. All authors critically reviewed the manuscript and approved the final version.

## Pre-publication history

The pre-publication history for this paper can be accessed here:

http://www.biomedcentral.com/1472-6874/13/21/prepub

## Supplementary Material

Additional file 1 User instructions of the first generation lavage device. Click here for file

Additional file 2 User instructions of the second generation lavage device. Click here for file

## References

[B1] Peto J Gilham C Fletcher O Matthews FE The cervical cancer epidemic that screening has prevented in the UK Lancet 2004 364 249 256 10.1016/S0140-6736(04)16674-9 15262102

[B2] Quinn M Babb P Jones J Allen E Effect of screening on incidence of and mortality from cancer of cervix in England: evaluation based on routinely collected statistics BMJ 1999 318 904 908 10.1136/bmj.318.7188.904 10102852 PMC27810

[B3] Van Ballegooijen M Van Den Akker Van M Patnick J Lynge E Arbyn M Anttila A Overview of important cervical cancer screening process values in European Union (EU) countries, and tentative predictions of the corresponding effectiveness and cost-effectiveness Eur J Cancer 2000 36 2177 2188 10.1016/S0959-8049(00)00330-0 11072201

[B4] Sigurdsson K The Icelandic and Nordic cervical screening programs: trends in incidence and mortality rates through 1995 Acta Obstet Gynecol Scand 1999 78 478 485 10.1080/j.1600-0412.1999.780602.x 10376856

[B5] Ronco G Giorgi-Rossi P Carozzi F Confortini M Dalla PP Del MA Efficacy of human papillomavirus testing for the detection of invasive cervical cancers and cervical intraepithelial neoplasia: a randomised controlled trial Lancet Oncol 2010 11 249 257 10.1016/S1470-2045(09)70360-2 20089449

[B6] Arbyn M Sanjose SD Saraiya M Sideri M Palefsky J Lacey C EUROGIN 2011 roadmap on prevention and treatment of HPV-related disease Int J Cancer 2012 131 1969 1982 10.1002/ijc.27650 22623137 PMC3429628

[B7] Rijkaart DC Berkhof J Rozendaal L van Kemenade FJ Bulkmans NW Heideman DA Human papillomavirus testing for the detection of high-grade cervical intraepithelial neoplasia and cancer: final results of the POBASCAM randomised controlled trial Lancet Oncol 2012 13 78 88 10.1016/S1470-2045(11)70296-0 22177579

[B8] Sasieni P Adams J Cuzick J Benefit of cervical screening at different ages: evidence from the UK audit of screening histories Br J Cancer 2003 89 88 93 10.1038/sj.bjc.6600974 12838306 PMC2394236

[B9] Bos AB Rebolj M Habbema JD van Ballegooijen M Nonattendance is still the main limitation for the effectiveness of screening for cervical cancer in the Netherlands Int J Cancer 2006 119 2372 2375 10.1002/ijc.22114 16858676

[B10] Bais AG van Kemenade FJ Berkhof J Verheijen RH Snijders PJ Voorhorst F Human papillomavirus testing on self-sampled cervicovaginal brushes: an effective alternative to protect nonresponders in cervical screening programs Int J Cancer 2007 120 1505 1510 10.1002/ijc.22484 17205514

[B11] Gok M Heideman DA van Kemenade FJ Berkhof J Rozendaal L Spruyt JW HPV testing on self collected cervicovaginal lavage specimens as screening method for women who do not attend cervical screening: cohort study BMJ 2010 340 c1040 10.1136/bmj.c1040 20223872 PMC2837143

[B12] Gok M van Kemenade FJ Heideman DA Berkhof J Rozendaal L Spruyt JW Experience with high-risk human papillomavirus testing on vaginal brush-based self-samples of non-attendees of the cervical screening program Int J Cancer 2012 130 1128 1135 10.1002/ijc.26128 21484793

[B13] Virtanen A Anttila A Luostarinen T Nieminen P Self-sampling versus reminder letter: effects on cervical cancer screening attendance and coverage in Finland Int J Cancer 2011 128 2681 2687 10.1002/ijc.25581 20669228

[B14] Virtanen A Nieminen P Luostarinen T Anttila A Self-sample HPV tests as an intervention for nonattendees of cervical cancer screening in Finland: a randomized trial Cancer Epidemiol Biomarkers Prev 2011 20 1960 1969 10.1158/1055-9965.EPI-11-0307 21752985

[B15] Wikstrom I Lindell M Sanner K Wilander E Self-sampling and HPV testing or ordinary Pap-smear in women not regularly attending screening: a randomised study Br J Cancer 2011 105 337 339 10.1038/bjc.2011.236 21730977 PMC3172898

[B16] Szarewski A Cadman L Mesher D Austin J Ashdown-Barr L Edwards R HPV self-sampling as an alternative strategy in non-attenders for cervical screening - a randomised controlled trial Br J Cancer 2011 104 915 920 10.1038/bjc.2011.48 21343937 PMC3065284

[B17] Giorgi RP Marsili LM Camilloni L Lossa A Lattanzi A Sani C The effect of self-sampled HPV testing on participation to cervical cancer screening in Italy: a randomised controlled trial (ISRCTN96071600) Br J Cancer 2011 104 248 254 10.1038/sj.bjc.6606040 21179038 PMC3031894

[B18] Holanda F Jr Castelo A Veras TM de Almeida FM Lins MZ Dores GB Primary screening for cervical cancer through self sampling Int J Gynaecol Obstet 2006 95 179 184 10.1016/j.ijgo.2006.07.012 16997304

[B19] Qiao YL Sellors JW Eder PS Bao YP Lim JM Zhao FH A new HPV-DNA test for cervical-cancer screening in developing regions: a cross-sectional study of clinical accuracy in rural China Lancet Oncol 2008 9 929 936 10.1016/S1470-2045(08)70210-9 18805733

[B20] Lazcano-Ponce E Lorincz AT Cruz-Valdez A Salmeron J Uribe P Velasco-Mondragon E Self-collection of vaginal specimens for human papillomavirus testing in cervical cancer prevention (MARCH): a community-based randomised controlled trial Lancet 2011 378 1868 1873 10.1016/S0140-6736(11)61522-5 22051739

[B21] Ogilvie GS Patrick DM Schulzer M Sellors JW Petric M Chambers K Diagnostic accuracy of self collected vaginal specimens for human papillomavirus compared to clinician collected human papillomavirus specimens: a meta-analysis Sex Transm Infect 2005 81 207 212 10.1136/sti.2004.011858 15923286 PMC1744976

[B22] Stewart DE Gagliardi A Johnston M Howlett R Barata P Lewis N Self-collected samples for testing of oncogenic human papillomavirus: a systematic review J Obstet Gynaecol Can 2007 29 817 828 17915065 10.1016/s1701-2163(16)32636-6

[B23] Petignat P Faltin DL Bruchim I Tramer MR Franco EL Coutlee F Are self-collected samples comparable to physician-collected cervical specimens for human papillomavirus DNA testing? A systematic review and meta-analysis Gynecol Oncol 2007 105 530 535 10.1016/j.ygyno.2007.01.023 17335880

[B24] Schmeink CE Bekkers RL Massuger LF Melchers WJ The potential role of self-sampling for high-risk human papillomavirus detection in cervical cancer screening Rev Med Virol 2011 21 139 153 10.1002/rmv.686 21538664

[B25] Brink AA Meijer CJ Wiegerinck MA Nieboer TE Kruitwagen RF Van KF High concordance of results of testing for human papillomavirus in cervicovaginal samples collected by two methods, with comparison of a novel self-sampling device to a conventional endocervical brush J Clin Microbiol 2006 44 2518 2523 10.1128/JCM.02440-05 16825374 PMC1489519

[B26] Dijkstra MG Heideman DA Van Kemenade FJ Hogewoning KJ Hesselink AT Verkuijten MC Brush-based self-sampling in combination with GP5+/6+−PCR-based hrHPV testing: high concordance with physician-taken cervical scrapes for HPV genotyping and detection of high-grade CIN J Clin Virol 2012 54 147 151 10.1016/j.jcv.2012.02.022 22445557

[B27] Belinson JL Du H Yang B Wu R Belinson SE Qu X Improved sensitivity of vaginal self-collection and high-risk human papillomavirus testing Int J Cancer 2012 130 1855 1860 10.1002/ijc.26202 21630255

[B28] Jolijn Coebergh Van Den B Corine V Albert H Daniëlle H Ben Willem M High acceptability of a modified lavage device for self-sampling Abstract IPV Conference Berlin 2012

[B29] Jones HE Brudney K Sawo DJ Lantigua R Westhoff CL The Acceptability of a Self-Lavaging Device Compared to Pelvic Examination for Cervical Cancer Screening Among Low-Income Women J Womens Health (Larchmt) 2012 21 1275 1281 10.1089/jwh.2012.3512 22906043

[B30] van den Brule AJ Pol R Fransen-Daalmeijer N Schouls LM Meijer CJ Snijders PJ GP5+/6+ PCR followed by reverse line blot analysis enables rapid and high-throughput identification of human papillomavirus genotypes J Clin Microbiol 2002 40 779 787 10.1128/JCM.40.3.779-787.2002 11880393 PMC120256

[B31] Hesselink AT Heideman DA Steenbergen RD Coupe VM Overmeer RM Rijkaart D Combined promoter methylation analysis of CADM1 and MAL: an objective triage tool for high-risk human papillomavirus DNA-positive women Clin Cancer Res 2011 17 2459 2465 10.1158/1078-0432.CCR-10-2548 21389098

[B32] Coupe VM Berkhof J Bulkmans NW Snijders PJ Meijer CJ Age-dependent prevalence of 14 high-risk HPV types in the Netherlands: implications for prophylactic vaccination and screening Br J Cancer 2008 98 646 651 10.1038/sj.bjc.6604162 18182990 PMC2243156

