# Supplementary material for: A second generation cervico-vaginal lavage device shows similar performance as its preceding version with respect to DNA yield and HPV DNA results
Source: BMC Womens Health. 2013 May 2;13:21. doi: 10.1186/1472-6874-13-21 (PMC3660212; doi:10.1186/1472-6874-13-21)
Supplement: Additional file 1 — User instructions of the first generation lavage device. [file 1472-6874-13-21-S1.pdf]

Introduction

Frequently screening can prevent women from getting cervical cancer by identifying signs of pre-cancer which can be treated. Just like the pap smear done in a clinic the *Pantarhei® Screener* can be used to screen for signs of risk for developing cervical cancer. The self-sampling test is quick, easy and painless.

The *Screener* is used to get a vaginal sample yourself. You gently insert the device, squirt a small amount of sterile water into your vagina, and collect the fluid back into the device. You can send your specimen in the pre-paid return envelope to . The investigator-doctor will contact you by telephone with the test result.

Please use the *Screener* within 2 weeks of receiving the kit. Before you proceed with the self-sampling you must have been found eligible for use by the Study Team and have signed the Informed Consent (Form 1).

Do not use the *Screener* when you are pregnant, while you are menstruating (during your period) or when you have never had sexual intercourse. If you use a vaginal product you need to stop using the product for 2 days before using the *Screener*. For more information about the *Screener* visit **www.pantarheiscreener.com**.

The screening will be conducted with care for your privacy. If you have any questions about using this home self-sampling test please contact the Study Team (see the back of this leaflet).

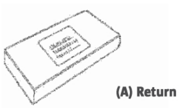

(A) Return box

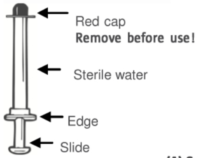

(A) Screener

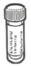

(A) Container

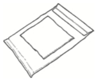

(A) Plastic bag and towel

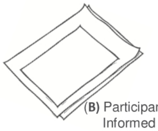

(B) Participant Information/
Informed consent (**Form 1**)
Questionnaire A+B (**Forms 2+3**)
Important Instructions

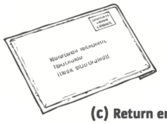

(C) Return envelope A and B

Step 1

Preparation

The package contains:
(A) Carton return box
(B) Study documents.
(C) Two return envelopes (A and B)

The container and forms 1,2 and 3 are labelled with a barcode. Please check that the number is the same on all three. If it is not, please contact the Study Team. You may not use the *Screener* beyond the expiration date that is printed on the blister of the *Screener*.

Before you proceed with self-sampling you are asked to fill in Questionnaire A (Form 2).

Wash your hands. Remove the cover of the container for your sample. Open the blister with the sterilized *Screener*. The device is long, but only about half of it will go in. There is a movable ring on the slide of the *Screener* which will not interfere with your use.

Lie down with a towel (as there may be some leakage) and pillow under your buttocks. Bend your knees with your legs more than shoulder distance apart. Take the *Screener*. **Hold the top up and remove the red cap**. Some water drops may be visible.

Step 2

The collection

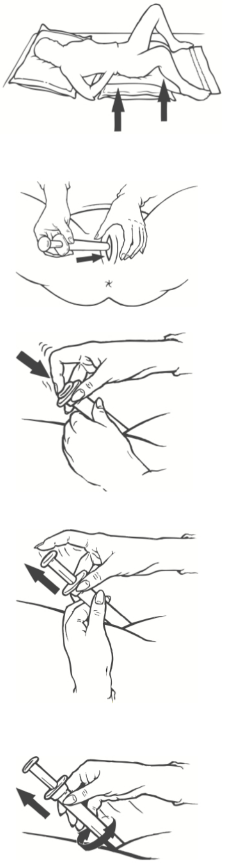

Hold the *Screener* with one hand near the edge and gently insert it with the round top as high as possible into your vagina until you feel some resistance.

Push the slide in slowly and completely in one motion, and hold it lightly in the down position while counting to 5. Then, release the slide to retrieve the fluid. The fluid will automatically be collected in the device.

Pull the *Screener* out of the vagina gently while turning it back and forth slightly as this will make the removal easier.

The *Screener* contains 5 ml of sterile water. As some water may leak from your vagina during this process, the sample will be less than 5 ml. This is normal. Do not worry, there will still be enough fluid collected to use for testing. In the rare case that there is not enough fluid the laboratory will contact you.

Step 3

Completion

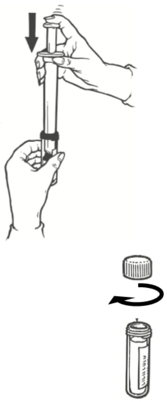

Sit up. Hold the opened container firmly in one hand. Hold the top of the *Screener* just inside the container. Empty the *Screener* by pushing the slide down. The fluid with your sample will flow from the *Screener* into the container. The sample will be cloudy and may be mucous; this is normal. Close the cover of the container firmly.

Wipe yourself off with a towel and get dressed. The *Screener* is for one-time use only. Throw the *Screener* away in the garbage. Wash your hands again.
