# Supplementary material for: A second generation cervico-vaginal lavage device shows similar performance as its preceding version with respect to DNA yield and HPV DNA results
Source: BMC Womens Health. 2013 May 2;13:21. doi: 10.1186/1472-6874-13-21 (PMC3660212; doi:10.1186/1472-6874-13-21)
Supplement: Additional file 2 — User instructions of the second generation lavage device. [file 1472-6874-13-21-S2.pdf]

## Read these instructions carefully before using the Delphi Screener

The Delphi Screener enables you to collect a fluid sample from your cervix and vagina in a simple and pain free way. It has been designed to make sure that the top will get close to the cervix with insertion in the vagina.

The top contains a small amount (1 teaspoon) of sterile water. By pressing the button at the back, the sterile water will spread around the cervical area. When the button is released, the water will flow back into the Screener with fluid from the cervix and vagina. After removing the Screener from the vagina the sample is then transferred into a test-tube. A laboratory will test the sample (please see enclosed return envelope for address details).

Do not use the Screener when you have your period (i.e. when menstruating). If you use vaginal medication or irrigation, you must stop using this 2 days before using the Screener. You can continue to use a vaginal ring, condoms or water-based lubricants. So far, there has been no experience of use with pregnant women, however due to the soft and rounded shape of the Screener, no health or safety risks are expected.

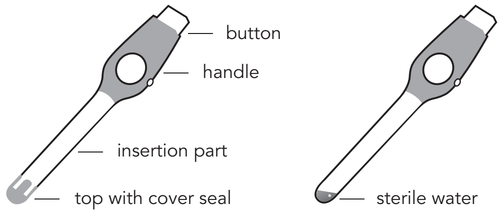


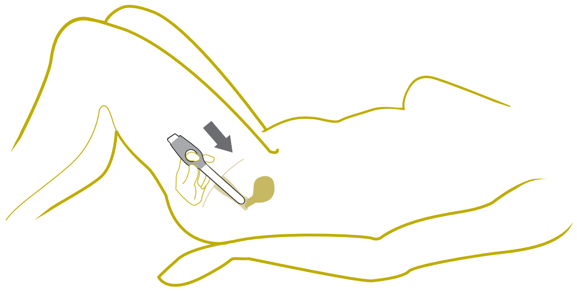

## Preparation

1. Check the content of the envelope:
   a - Study Documents
   b - Return Envelope
   Cardboard box containing:
   c - Delphi Screener
   d - Test-tube
   e - Plastic bag with absorbent pad
   f - User Instructions

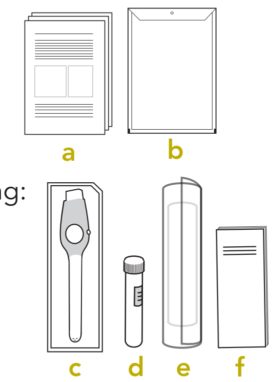


Please check that your **personal identification number** on the test-tube and on the Consent Forms match. Complete and sign both Consent Forms.

2. Wash your hands.

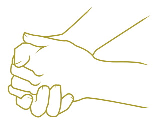

3. Take out the test-tube, remove the cap and keep both nearby.

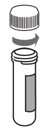

4. Open the pouch with the Screener until it is completely open.

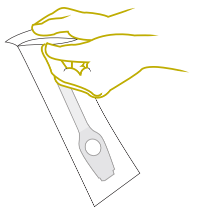

5. Take the Screener by holding the blue handle.

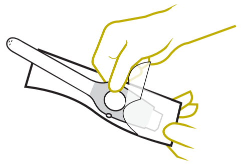

6. Pull the cover seal off the top of the Screener. Some water drops may be visible.

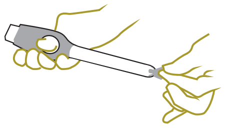

## Choose your position

7. **a. Lying down**
   Cover a pillow with a towel. Place your bottom on the pillow. Bend your knees and spread your legs.

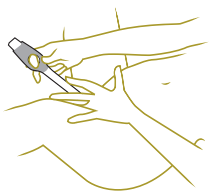

   **b. Sitting down**
   Sit on the toilet seat with your legs apart.

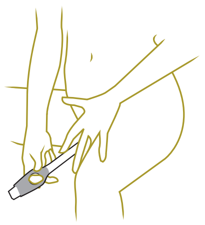

## How to use the Screener?

You may notice some fluid coming out of your vagina after obtaining the sample. This is not a problem. Only a small amount of fluid in the test-tube is needed for the test. You may find some blood or mucus on the Screener – this is normal and will not influence the results of your test.

8. Hold the Screener by the blue handle as shown in the picture and use your other hand to spread your labia (vaginal lips). Insert the top of the Screener slowly and gently into the vagina, tilting it backwards, as deep as possible until you feel resistance.

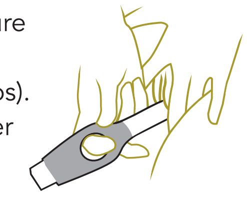

9. Hold the Screener whilst pressing the white button fully down.

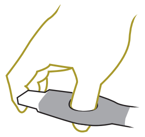

10. Once the button is fully down, count to 3.

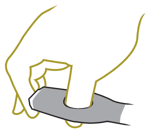

11. Let go of the button and take the Screener out of your vagina **slowly**, turning it as you do so. The flushed fluid will then flow back into the screener.
    Sometimes the white button does not return immediately to its initial position when taking the Screener out. This is OK.

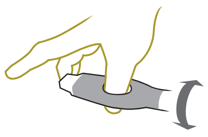

12. Insert the Delphi Screener into the test-tube with the top halfway down. Keep the tube and Screener in an upright position. Press the button of the Screener fully down. The sample fluid will then be released into the tube. The sample will look cloudy and may contain mucus; this is normal.

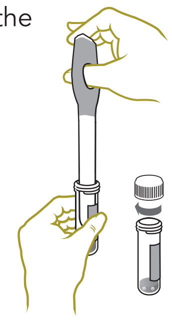

13. Take the Screener out of the tube. Place the cap on the tube and make sure it is screwed tightly.

## What to do after taking a sample?

14 The Screener can only be used once.
Throw the Screener away in a rubbish bin.

15 Wash your hands again.

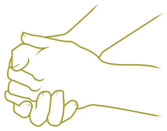

16 Put the test-tube in the plastic bag with absorbant pad. Close the bag with the seal strip.

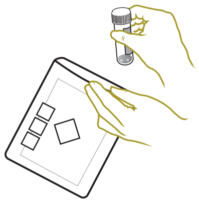

17 Return the plastic bag containing the test-tube into the cardboard box, together with one completed and signed Consent Form, and close the box.

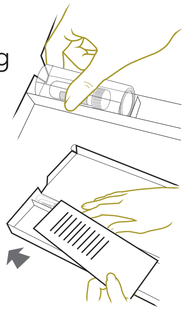

18 Put the box into the return envelope. **Retain the other Consent Form for your information.**
The return envelope is addressed and the postage has been paid.
You can mail it without a stamp.

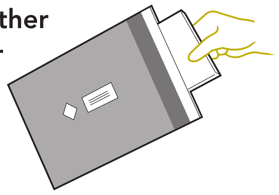

19 Your test result will be available in 4 weeks from the date your sample is received in the laboratory. You will have indicated on the Consent Form how you prefer to be contacted with your test result (text, letter, phone).

If you have any questions about your result, or if you have not received your result after 4 weeks please contact the research team (see the Patient Information Sheet for contact details).

**RETURN YOUR SAMPLE WITHIN 24HRS AFTER COLLECTION TO GUARANTEE THE QUALITY OF THE SAMPLE!**

## General information

You cannot use the Screener after its best before date. This is indicated on the pouch (⌛..-....).

If the pouch is damaged or opened, the sterility of the Screener cannot be guaranteed and a new one must be used.

Please visit www.delphiscreener.com for an animation video on the use of the Delphi Screener, for additional information on the Screener or for User Instructions in other languages.

STERILE R

5°C 40°C

Delphi Bioscience B.V.
Glashorst 126
3925 BV Scherpenzeel
The Netherlands

CE 0344

Date of last revision: June 2012 -210033-

**Disclaimer**
Delphi Bioscience cannot accept responsibility in any way for careless or incorrect use of the medical device. If, after reading these instructions, there are any questions or doubts about the use of the device, please contact the research team.



## User Instructions Delphi Screener

delphi bioscience
